# Biomonitoring and Health Risk Assessment of Arsenic Contamination in Drinking Water among Rural Residents in Western Tehran

**DOI:** 10.1371/journal.pone.0317527

**Published:** 2025-02-13

**Authors:** Motahareh Harati, Seyed Mohammad Tabatabaei Jabali, Yousef Abdossalami Asl, Mahdi Chinichian, Tahereh Donyavi, Niloufar Bahari, Hadi Jalilvand, Negin Kassiri, Zahra Asadgol

**Affiliations:** 1 Health Deputy, Iran University of Medical Sciences, Tehran, Iran; 2 Department of Sport and Exercise Medicine, School of Medicine, Iran University of Medical Sciences, Tehran, Iran; 3 Reference Laboratory, Health Deputy, Iran University of Medical Sciences, Tehran, Iran; 4 Occupational Medicine Research Center, Iran University of Medical Sciences, Tehran, Iran; 5 Department of Occupational Medicine, School of Medicine, Iran University of Medical Sciences, Tehran, Iran; SKUMS: Shahrekord University of Medical Science, ISLAMIC REPUBLIC OF IRAN

## Abstract

Arsenic is a widespread environmental contaminant that poses a significant threat to global health due to its toxicity and carcinogenicity. Given the high levels of arsenic found in the drinking water of western areas of Tehran, the objective of this study was to analyze levels of arsenic in multiple biological samples (blood, hair, and nails) collected from residents living in these areas. This cross-sectional study was conducted over three weeks in November 2022 in five villages. A total of 67 residents from these villages were included in the exposure group. Analysis of arsenic was carried out by using the Perkin Elmer Optima 8000 ICP-OES instrument coupled with the FIAS 100 flow injection module after sample digestion. The average concentration of arsenic in people’s blood was 4.19 μg/l, which exceeds the standard limit of ATSDR (1 μg/l) by about 4 times. Additionally, 47.8% of blood samples exceeded the standard, while for nail and hair samples, the percentages were 22.4% and 13.4%, respectively. Water samples showed the highest percentage above the standard, with 67.2%. There is no significant relationship between arsenic levels in drinking water, hair and blood. However, a significant positive correlation was observed between the concentration of arsenic in drinking water and nail samples. The mean of hazard quotient (HQ) and carcinogenic risk (CR) indices of arsenic in drinking water suggest that the daily intake levels of the examined arsenic in the study area exceeded the acceptable thresholds ((HQ < 1) and (CR < 1 × 10^−4^)). Although this study demonstrated elevated arsenic exposure among the population in western Tehran, our findings showed no significant correlation between arsenic concentrations in drinking water and biological samples. Therefore, further research is required to identify other potential exposure pathways and develop targeted intervention strategies. Additionally, remediation measures to improve water quality remain essential in this rural area.

## 1. Introduction

Arsenic, a metalloid, is naturally present in soil, atmosphere, natural waters, and organisms undergoes mobilization through a blend of natural processes, including weathering reactions, biological activity, and volcanic emissions, as well as a variety of human-induced activities [1]. The majority of environmental arsenic issues are due to its mobilization occurring under natural conditions. Total arsenic is composed of both inorganic and organic species. Organic arsenic is considered not or less toxic to humans [2]. In contrast, the toxic inorganic forms, arsenate (AsV) and arsenite (AsIII) are prevalent in both drinking water and various food sources, including rice, non-rice grains, vegetables, fruits, meats, dairy products, and seaweed [3,4]. The primary contributor of inorganic arsenic to the diet is contaminated water, with ingestion through food—especially rice—serving as another significant exposure pathway. However, in some regions of the world, arsenic exposure through contaminated drinking water is a substantial concern compared to other exposure pathways [5,6]. Consequently, the World Health Organization (WHO) has established a guideline for arsenic concentration in drinking water at 10 μg/liter [7]. More than 250 million individuals in about 108 countries globally rely on arsenic-contaminated groundwater for their drinking water supply [8].

Arsenic toxicity and mechanism of action are complex and multifaceted. The main pathways through which arsenic exerts health effects include oxidative stress, DNA damage and repair inhibition, epigenetic alterations, enzyme inhibition, disruption of cellular signaling pathways, and apoptosis induction [9–11]. According to the International Agency for Research on Cancer (IARC) reports, ingestion of inorganic arsenic has a causal role in skin, bladder, lung, liver, and kidney cancers [12]. Furthermore, chronic ingestion of arsenic is linked to other adverse health effects including cardiovascular disease, skin lesions, developmental toxicity, abnormal glucose metabolism, neurotoxicity, and diabetes [13,14]. However, the toxic effects of arsenic may vary depending on factors such as gender, age, life stage, nutritional status, and others [15]. While some epidemiological studies have suggested a potential association between maternal arsenic exposure during pregnancy and adverse birth outcomes, including infant mortality, low birth weight [16], gestational diabetes (GDM) [17], preterm birth, and preeclampsia [18], the evidence remains inconclusive. Additional research is required to confirm these associations and to understand the underlying mechanisms. Different studies in various regions also demonstrated statistically significant associations between lung cancer and drinking water with high concentrations of arsenic [19,20]. In a prospective cohort study conducted in Bangladesh, Yu Chen and her team contributed further evidence supporting the association between arsenic in water and an elevated risk of mortality from cardiovascular disease [21].

As a result of growing pollution, the population may be exposed to chemicals via multiple pathways [22]. Human biomonitoring (HBM), is known as a useful method for identifying health effects of environmental pollution by direct measurement of chemicals and their metabolites in human tissues and fluids [23]. Human biomonitoring can recognize specific at risk groups in high environmental pollution exposures and then be used to intervene in public health challenges [24]. Eventually, it is a powerful method for following the effectiveness of interventions. Considering, the utilities of human biomonitoring to carry out an integrated estimation of environmental exposure, HBM national programs established by some countries to progress the detection, treatment, prevention and harmful environmental exposures in populations [22].

The results of routine sampling of the rural network water system several months prior in some villages in western Tehran indicated arsenic concentrations exceeding standard safety limits, highlighting the need for further investigation. These findings prompted us to design a biomonitoring study aimed to assess the health effects of arsenic exposure on residents in the affected area. Consequently, due to the previous contamination, a cross-sectional human biomonitoring study was conducted to analyze arsenic levels in hair, nail, and blood samples from people living in this region.

## 2. Materials and methods

### Ethics approval and consent to participate

The Human and Animal Research Ethics Committee of Iran University of Medical Sciences approved all experimental procedures in the current study (ethical code: IR.IUMS.REC.1401.589). The experiment was performed with respect to the guidelines of the Specific National Ethics for Biochemical Research issued by the Research and Technology Deputy of the Ministry of Health and Medical Education (MOHME) of Iran (issued 2005). Researchers provided participants with information about the study. Once participants are convinced and agree to participate, they are asked to sign a written informed consent form. In addition, samples were gathered from minors and/or children after obtaining their parents’ or guardians’ written informed consent. All selected participants were informed about the method of sampling in face-to-face interviews.

### 2.1. Sampling location

**Fig 1:**
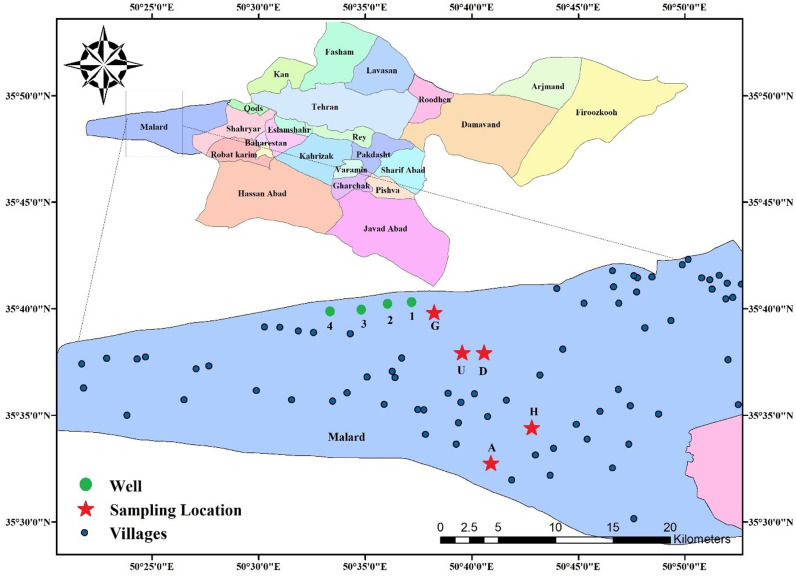
The study area and sampling locations; West of Tehran province, Iran (Created by Arc GIS version 10.2).

Tehran (35° 41 ‘21 ‘N, 51° 23′ 20’ E), the capital of Iran, covers an area of 730 km^2^. Its population stands at approximately 8.4 million permanent residents, while the daily influx reaches 15 million individuals because of commuters from the surrounding regions (Fig 1). Tehran has notable variations in weather conditions across diverse districts. The lowest daily temperature is in January (−15°C) and the highest occurs in July (43°C) with a daily average annual temperature of 18°C [25]. The studied area is located in the west of Tehran with 23 villages. The only source of drinking water in this region is underground water sources (drilled wells). In terms of climate, this region is considered one of the hot and dry regions, while strong cold and dry winds in winter and hot and dry winds with dust in summer are among the climatic characteristics of this region.

This region is situated in the Urmia-Dokhtar volcanic belt which is considered one of the potentially hazardous regions in terms of heavy metal pollution due to its geological structure. The Urmia-Dokhtar magmatic arc is primarily composed of andesite and dacite. In this region, arsenic is notably observed in common rock-forming minerals of trachyte, basalt trachyte, dacite, sinodiorite dacite, and basaltic dikes. Furthermore, this region is notable for the mineralization of various elements including gold, silver, copper, lead, zinc, iron, manganese, tin, and tungsten. As these elements are commonly associated with arsenic, it emphasizes the enrichment of rock with arsenic [26].

### 2.2. Sample collection

This cross-sectional study was conducted for 3 weeks in November 2022 in five villages of G, U, D, A, and H. A total of 67 residents were considered as the exposure group. The samples were selected using different sampling methods depending on the population size of the villages to ensure a representative and comprehensive analysis. In villages with larger populations, a random sampling method was employed to ensure that each resident had an equal chance of being included in the sample set. A comprehensive list of all residents in these villages was compiled, with each resident assigned a unique identification number. Participants were then randomly selected from this sampling frame using a random number generator. In villages with smaller populations, where the number of residents was low, all available participants were included in the sample set. This approach ensured that the sample size was sufficient for reliable statistical analysis and that the entire population was adequately represented. After sample selection, a checklist was designed to note the demographic information and intervention factors of each individual. In this checklist, some information such as age, sex, nationality and occupation, smoking status, use of pesticide and consumption of seafood, use of hair removal compounds, use of nail polish and hair dye, type of rice consumed, and type of water consumed and more was recorded. Researchers provided participants with the necessary information about the study. Once participants are convinced and agree to participate, they are asked to sign an informed consent form. Sample selection entry criteria were residents of villages in Western Tehran, users of rural network water for various consumption purposes, and also provided informed consent to partake in the study. Criteria for excluding samples from the study were samples with incomplete demographic or health information, participants who did not follow study protocols, and samples that could not be accurately analyzed due to contamination or other technical issues.

The rationale for using blood, hair, and nail samples to assess arsenic exposure lies in their distinct properties, each offering complementary information that enhances our understanding of exposure levels. Blood is a useful indicator of recent arsenic exposure while hair and nail samples reflect long-term arsenic exposure. Participants were instructed to remove nail polish before sample collection to avoid contamination. All selected participants were informed about the method of sampling in face-to-face interviews and then they were asked to cut their nails from every 10 fingers using steel nail clippers. For hair samples, dyed hair was noted, and participants were asked about the timing and frequency of hair dye use. If recent dyeing was reported (within the last month), those samples were noted as potentially influenced by the dye. Then newly grown hair samples were taken from the back of the head (around the neck) or behind the ears, close to the scalp with 3–5 cm length, with stainless steel scissors that was specially made for hair cutting. Scalp hair and nail samples were packed separately in labeled polyethylene zip-lock bags [6]. Blood was also collected by the sampler in 7 ml royal blue tubes with EDTA anticoagulant and stored at 4°C during transportation and at −20°C until processing and analysis in the laboratory [27]. Also, researchers asked participants to provide samples of water used in their daily lives, both for drinking and cooking. This could include tap water, water from purification devices (such as filters or reverse osmosis systems), bottled water, or any other sources of water they regularly use. Water samples were collected in pre-cleaned polypropylene containers transported to the laboratory and kept at room temperature until analysis [6].

### 2.3. Laboratory analysis

#### 2.3.1. Water samples analysis.

All arsenic measurements were performed using ICP-OES instruments coupled with the FIAS sample preparation method to improve the detection limit of arsenic in samples. The water samples were acidified to pH = 2.0 and stored at room temperature for future analysis according to the EPA method No. 200.7 [28].

#### 2.3.2. Biological samples analysis.

To digest and measure the arsenic, around 300 mg of hair sample, and 30 mg of fingernail clippings were applied. The nail samples were washed with acetone and ultrapure water in an ultrasonic bath to remove external interference compounds. This washing process was done in 5 steps. At first, the samples were washed with acetone. Then, in the next three steps washing was performed with double-distilled water, and at the end, samples were washed with acetone. The sonication in each step was done for 5 minutes. After washing processes, the samples were dried in an oven at 60°C for 2 hours [29]. Hair samples were washed with baby shampoo in the laboratory to remove interference dust and particles. Hair samples washing steps were done like nail samples to remove contamination. Then, the hair samples were cut into 5 mm pieces [30]. All biological samples (including blood, hair, and nails) were digested by Milestone microwave digestion (Rom, Italy) according to the method suggested by the application note of the device by adding 65% nitric acid and H_2_O_2_ reagents. In this section, we used 4 ml HNO_3_ and 1 ml H_2_O_2_ (4:1) for digestion of biological samples. Whole blood samples were homogenized before digestion. Approximately, 1 ml of blood sample was used for the measurement of arsenic. Analysis of arsenic was carried out by using the PerkinElmer Optima 8000 Dual View ICP-OES instrument coupled with a FIAS 100 hydride generation system (Shelton, Connecticut, USA). Moreover, spectrophotometric data were recorded and analyzed using Syngistix for ICP (version 2.0.0.2236), developed by PerkinElmer. Sensitivity and lower detection limits, reduced costs, higher speed and efficiency, as well as interference reduction, are the advantages of ICP-OES compared to other analytical methods. These features make it a more suitable choice for the precise and comprehensive analysis required in our study [31]. The calibration curve was constructed using standard arsenic solutions prepared from a certified stock solution. We prepared a series of standard solutions with concentrations ranging from 0.3 ng/mL to 10 ng/mL. Calibration standards were diluted from the stock solution with the appropriate sample matrix, and each standard was analyzed in triplicate to ensure accuracy. The calibration curves were linear in the range of 0.3–10 ng/mL, with a coefficient of determination (R²) higher than 0.9952, indicating excellent linearity. The detection limit (LOD) and quantification limit (LOQ) values were 0.1 ng/mL and 0.3 ng/mL, respectively.

### 2.4. Health risk assessment

According to the United States Environmental Protection Agency (USEPA), exposure to heavy metals such as arsenic (As) through drinking water can pose significant health risks to humans. Therefore, in the current study, the initial assumption is that arsenic may potentially lead to adverse health effects, including both carcinogenic (cancer-causing) and non-carcinogenic risks via the consumption of drinking water in the study area.

#### 2.4.1. Exposure assessment.

In this study, risk assessment for arsenic was assessed based on average daily dose (ADD) and hazard quotient (HQ) via drinking water. The ADD was calculated using the following formula, which was adapted from the US EPA 2007 [32–34].


$$\text{ADD=}\left(\text{C×IR}\right)\text{/}\left(\text{BW}\right)$$

Where ADD is expressed as an average daily dose of arsenic (μg kg^−1^ day^−1^), C is the concentration of arsenic (μgL^− 1^), IR is the daily water ingestion rate (L day^−1^), which its average consumption rate for Iranian adults is 2 L/day [33]. BW also is body weight of 70 kg for adult groups [33].

#### 2.4.2. Non-carcinogenic risk (HQ) assessment of arsenic.

The hazard quotient (HQ) is calculated by dividing the estimated exposure dose of arsenic by the reference dose (RfD). The HQ provides a numerical estimate of the potential risk of adverse health effects from exposure to arsenic. If the HQ is greater than 1, it indicates that the estimated exposure dose exceeds the reference dose or concentration, suggesting a potential risk of adverse health effects. While the exposed population is deemed safe if the Hazard Quotient (HQ) is less than 1 [32,35].


HQ=ADD/RfD

RfD is the oral reference dose to indicate “The daily exposure level that the human population can expose over a lifetime without significant adverse effects”. RfD for arsenic is 0.0003 mg kg^ −1^ day^−1^ [33].

#### 2.4.3. Carcinogenic risks (CR) assessment of arsenic.

According to the EPA definition, carcinogenic risk is “the incremental risk of an individual developing cancer over their lifetime due to exposure to a potential carcinogen” [36]. Carcinogenic Risks was calculated using the following formula:


CR=ADD×CSF

Where CSF is the cancer slope factor for arsenic of 1.5 mg kg^−1^ day^−1^ [37]. CR is known as a risk level of 1 × 10^−6^ and is regarded as the threshold for excess cancer risk, signifying a 1 in 1,000,000 chance of developing cancer through the consumption of drinking water containing arsenic (μg/L) over 70 years. The safe threshold for carcinogenic risks should be below this level. The range of risks borderline by the EPA is 1 × 10^−4^ to 1 × 10^−6^. While CR more than 1 × 10^ − 4^ is unacceptable and poses health hazards [38].

### 2.5. Statistical analysis

The statistical package SPSS version 21 and Excel 2016 (Microsoft Office) were used to calculate descriptive statistics. The kolmogorov-smirnov test was done for the evaluating the normality of the data. The comparison of average arsenic concentrations in each medium and independent variables was performed through the Independent Samples T-Test. The Pearson correlation coefficient was applied to analyze the correlation between arsenic in drinking water and arsenic in blood, hair, and nails. The level of significance was defined as α = 0.05 and a value of p <  0.05 was indicative of statistical significance. Moreover, for designing the map of the study area and sampling locations, ArcMap 10.2 Geographical Information System (GIS) (ESRI, Redlands, CA) was carried out.

## 3. Result

### 3.1. Characteristics of study participants

Out of the 67 distributed questionnaires, all questionnaires were usable. The average age of the subjects studied was 43.3 ±  18.7 years (with an average age of 42.7 years for men and 44 years for women). The highest age examined was 90 years, and the lowest age was 11 years. Table 1 shows the average, minimum, and maximum age of people living in the region.

**Table 1:** Age characteristics of study participants.

Sex	Age range	Mean (SD)
Male	11–90	42.72 (20.4)
Female	11–71	44.00 (16.5)
Both	11–90	43.37 (18.7)

Table 2 also provides an overview of the general characteristics of the subjects under study, through the results of the completed checklists. According to the findings presented in this table, 49.3% of the participants were male, while 50.7% were female. Of these individuals, 89% were Iranian, with the remaining being non-Iranian. The highest level of participation in terms of occupational status was observed among housewives, constituting 30 individuals (44.8%). Regarding the use of agricultural pesticides, 55 participants opted not to use them (82.1%). Furthermore, 11.9% of the participants reported using hookah, 10.4% smoked, and 5.9% used drugs. Among the 67 participants in this research, 28.3% sourced their drinking water from the rural water supply network with possessing a home water purifier. While, 62.7% of the participants relied solely on the rural water supply network without a purifier for their water needs, and only 9% purchased purified water from authorized stores for their consumption. Additionally, the proportions of participants who were consumers of seafood, depilatory powder, individuals using nail polish or nail implants, and those using hair dye were 19.4%, 25.4%, 13.4%, and 37.3%, respectively. Furthermore, the majority of participants (39 individuals, 58.2%) exclusively consumed Iranian rice.“

**Table 2:** General characteristics of the participants.

Characteristics		No.	Percent (%)
Subjects number		67	100
Gender	Male	33	49.3
	Female	34	50.7
Nationality	Iranian	60	89.6
	Afghan*	7	10.4
Occupation	Driver	3	4.5
	Housewife	30	44.8
	Office worker	3	4.5
	Rancher	13	19.4
	Seller	3	4.5
	Student	5	7.5
	Welder	3	4.5
	Manual worker	6	9
	Unemployed	1	1.5
Smoking status	Hookah	8	11.9
	Cigarette	7	10.4
	Drugs	4	5.9
	None	51	67.1
Pesticide use	Yes	13	19.4
	No	54	80.6
Seafood consumption	Yes	13	19.4
	No	54	80.6
Using hair removal powder	Yes	17	25.4
	No	50	74.6
Nail polish and nail implants	Yes	9	13.4
	No	58	86.6
Use of hair dye	Yes	25	37.3
	No	42	62.7
Herbal medicines consumption	Yes	12	17.9
	No	55	82.1
Type of rice consumed	Domestic	39	58.2
	Imported	11	16.4
	Both	17	25.4
Type of drinking water consumed	Rural network with household water purifier	19	28.3
	Rural network without household water purifier	42	62.7
	Purchased purified water	6	9.0

*They were a family members and have migrated to this region since almost ten years ago

### 3.2. Arsenic in the blood, hair, nails, and water of the participants

As shown in Table 3, the average, standard deviation, maximum, and minimum arsenic values in blood, hair, nail, and water samples are presented. According to the CDC’s Agency for Toxic Substances and Disease Registry (ATSDR), the reference values for arsenic concentration in blood is 1 μg/l [39,40], while the average concentration of arsenic in the participant’s blood was 4.19 μg/l, which exceeds the standard limit by about 4 times. The average concentration is higher in men (4.52 μg/l). The highest recorded concentration of arsenic was found in the blood of a 28-year-old man with a value of 15.93 μg/l. Similarly, the average concentration of arsenic in men’s hair is higher, around 0.48 μg/g, which is below the standard (1 μg/g) [41]. The highest concentration of arsenic was recorded in the hair of a 37-year-old man, approximately 4.87 μg/l. Furthermore, the results of this study indicate that the average concentration of arsenic in nails, for both men and women, exceeds the standard limit (1 μg/g) [41], measuring about 2.82 and 3.06 μg/g, respectively. The highest concentration of arsenic in fingernails was detected in a 28-year-old Afghan man, surpassing the standard limit by approximately 77 times. The average concentration of arsenic in drinking water was recorded as 15.8 μg/l, exceeding the standard limit by about 1.5 times (10 μg/l). Meanwhile, the maximum recorded arsenic concentration was 54.36 μg/l.“

**Table 3:** Arsenic levels in the blood, hair, nails and water samples of the participant by gender.

Arsenic	Unit	Sex	Min	Max	Mean (SD)
**In blood**	**µg/l**	**Male**	0	15.93	4.52 (5.21)
		**Female**	0	14.14	3.87 (5.1)
		**Both**	0	15.93	4.19 (5.2)
**In hair**	**µg/g**	**Male**	0	4.87	0.48 (1.18)
		**Female**	0	3.03	0.31 (0.66)
		**Both**	0	4.87	0.39 (0.96)
**In nail**	**µg/g**	**Male**	0	77.33	3.06 (13.3)
		**Female**	0	27.92	2.82 (6.5)
		**Both**	0	77.33	2.94 (10.5)
**In water**	**µg/l**	**Male**	0	54.36	15.87 (12.3)
		**Female**	0.02	41.46	15.72 (11.4)
		**Both**	0	54.36	15.8 (11.92)

**Fig 2:**
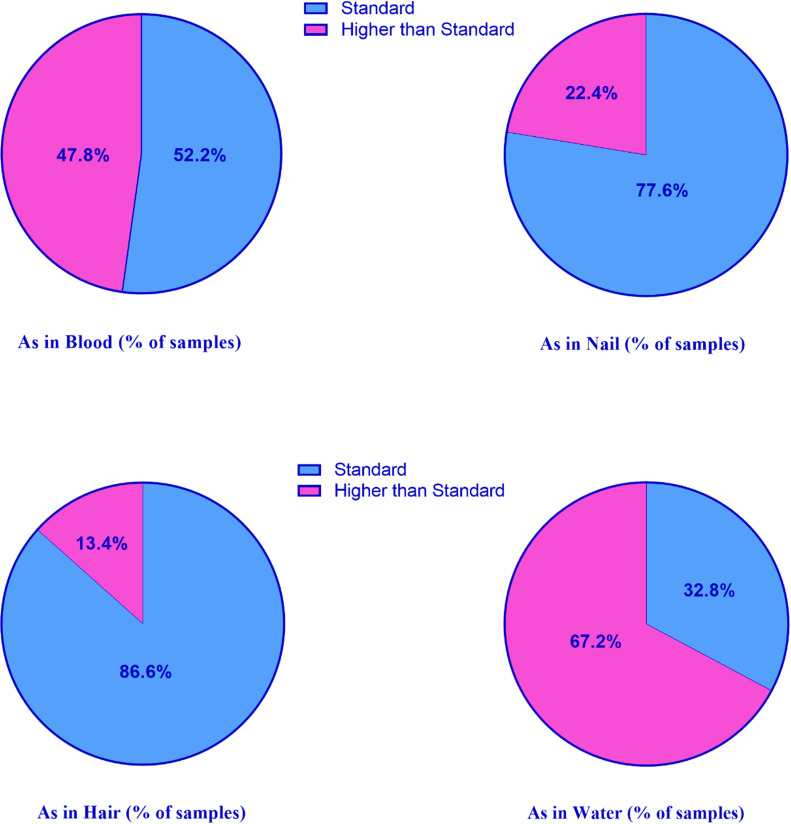
The presence of arsenic in the samples compared to the standard value.

Fig 2 illustrates the percentage of samples surpassing the standard across various sampling media. As depicted, 47.8% of blood samples exhibit values exceeding the standard, whereas for nail and hair samples, these percentages are 22.4% and 13.4%, respectively. Upon scrutinizing the samples, it was found that the highest number of samples exceeding the standard was related to water samples (46 samples, constituting 68.6%). Of these, 76% were related to rural network without household water purifier, and 73% were related to rural network with household water purifier. All the Purchased purified water samples had arsenic levels below the standard limit.

### 3.3. Arsenic concentration in samples by village

**Fig 3:**
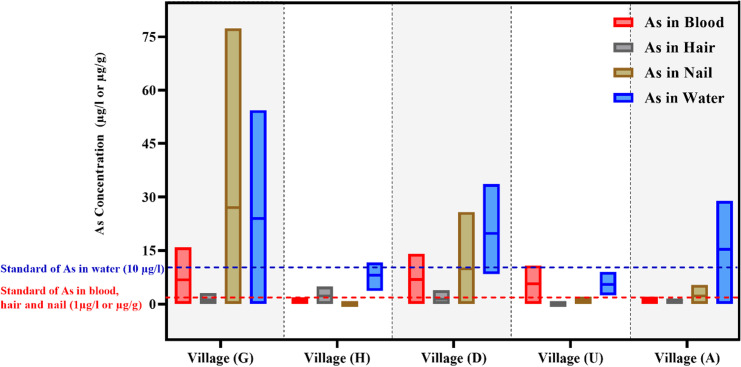
The range of arsenic concentration in blood, hair, nail and water samples of participants by village.

Fig 3 presents the range of arsenic concentrations in blood, hair, nail, and water samples collected from different villages. As illustrated below, the arsenic concentration in water samples from village G ranged from 0.02 to 54 μg/l, whereas in village H, it ranged between 3 and 11 μg/l. The average arsenic concentration in water from village D is approximately 17.5 μg/l (ranging from 8.3 to 33.6 μg/l). Notably, in village U, all water samples had arsenic concentrations below the standard limit, measuring between 2 and 9 μg/l (average of 5 μg/l). Conversely, in village A, the range was between 0 and 29 μg/l, with an average concentration of 17 μg/l.

In terms of nail samples, arsenic concentrations in villages U and H were reported below the standard limit. However, in village G, concentrations ranged from 0 to 77 μg/g, with an average of 4 μg/g. Following G village, the highest concentration was observed in village D, ranging from 0 to 26 μg/g. The highest concentrations of arsenic in blood samples were detected in villages G (ranging from 0 to 16 μg/l) and D (ranging from 0 to 14 μg/l). Conversely, arsenic levels in villages H and A varied between 0 and 2 μg/l.

**Fig 4:**
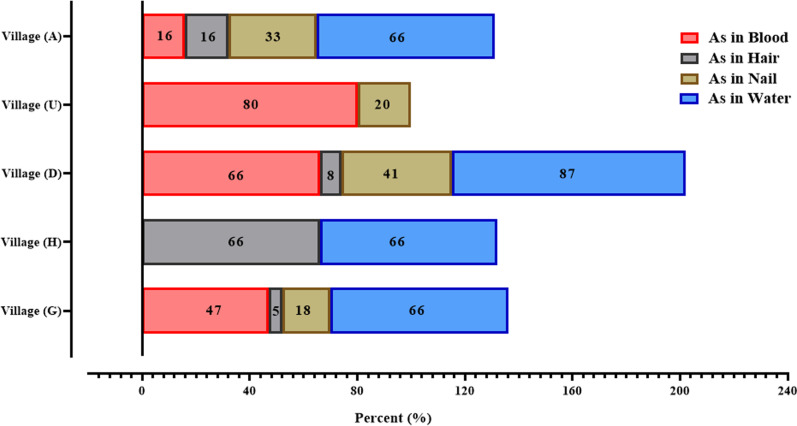
Percentage of samples with arsenic concentration higher than standard by village.

Fig 4 depicts the percentage of samples with arsenic concentrations exceeding the standard across villages G, H, D, U, and A. The village D exhibited the highest percentage of water samples surpassing the standard limit (10 μg/l), with 87% of cases. Conversely, none of the water samples from Village U exceeded the standard. Furthermore, Village D also recorded the highest percentage of nail samples with arsenic concentrations above the standard, at 41%. Additionally, the villages U and H demonstrated the highest percentages of blood and hair samples exceeding the standard, with 80% and 66%, respectively.

### 3.4. Relationship between arsenic concentration of drinking water and biological samples

The differences in the average arsenic concentrations in blood, hair, nails, and water based on various independent variables were examined using an independent t-test, with results presented in Table 4. According to the table, blood arsenic concentrations differed significantly between smokers and non-smokers (p-value =  0.002) and between those who had undergone nail planting and those who had not (p-value =  0.003). Additionally, in the sampling region, significant differences in hair arsenic concentration were observed between participants based on cigarette and hookah use, seafood intake, use of hair removal powder and hair dye, and family history of addiction. Nail arsenic concentrations also showed significant differences between those who consumed seafood, those who consumed Iranian rice, and participants with a family history of addiction.

**Table 4:** The average amount of arsenic in blood, hair and nails within independent variables (using Independent Samples T-Test).

	variable		No.	Mean of blood arsenic	Mean of hair arsenic	Mean of nail arsenic
**1**	Gender	Male	33	4.81	0.48	3.05
		Female	34	4.24	0.30	2.82
		**p-value**	**–**	0.80	0.07	0.65
**2**	Pesticide use	**No**	54	4.30	0.43	3.46
		**Yes**	13	5.36	0.23	0.77
		**p-value**	–	0.83	0.23	0.15
**3**	Cigarette smoking	**No**	60	4.90	0.44	3.19
		**Yes**	7	1.55	0.00	0.79
		**p-value**	–	**0.002**	**0.02**	0.34
**4**	Hookah smoking	**No**	59	4.41	0.31	3.06
		**Yes**	8	5.60	0.95	2.04
		**p-value**	–	0.82	**0.009**	0.62
**5**	Seafood consumption	**No**	54	4.38	0.26	2.09
		**Yes**	13	5.48	0.95	6.43
		**p-value**	–	0.51	**0.004**	**0.006**
**6**	Using hair removal powder	**No**	48	4.51	0.50	3.18
		**Yes**	19	4.57	0.07	2.21
		**p-value**	–	0.79	**0.001**	0.46
**7**	Nail polish	**No**	61	4.45	0.39	3.22
		**Yes**	6	5.65	0.43	0.00
		**p-value**	–	0.38	0.88	0.22
**8**	Nail implants	**No**	64	4.68	0.38	2.39
		**Yes**	3	0.00	0.64	2.97
		**p-value**	–	**0.003**	0.56	0.90
**9**	Use of hair dye	**No**	42	4.25	0.53	3.06
		**Yes**	25	5.00	0.16	2.72
		**p-value**	–	0.18	**0.001**	0.50
**10**	Domestic rice consumed	**No**	11	6.28	0.24	8.62
		**Yes**	56	4.15	0.42	1.82
		**p-value**	–	0.22	0.56	**0.04**
**11**	Imported rice consumed	**No**	39	4.73	0.54	1.38
		**Yes**	28	4.27	0.18	5.10
		**p-value**	–	0.73	0.13	0.15
**12**	Household water purifier	**No**	50	4.41	0.18	4.10
		**Yes**	17	4.90	0.91	0.00
		**p-value**	–	0.48	**0.001**	**0.018**
**13**	Water boiling before consumed	**No**	61	4.16	0.43	2.60
		**Yes**	6	7.89	0.00	6.34
		**p-value**	–	0.93	**0.033**	0.58
**14**	Drug user in family	**No**	54	4.63	0.45	1.93
		**Yes**	13	4.12	0.15	7.12
		**p-value**	–	0.47	**0.05**	**0.004**
**15**	Herbal medicines consumption	**No**	55	4.76	0.32	3.44
		**Yes**	12	3.16	0.73	0.63
		**p-value**	–	0.19	**0.044**	0.15

**Fig 5:**
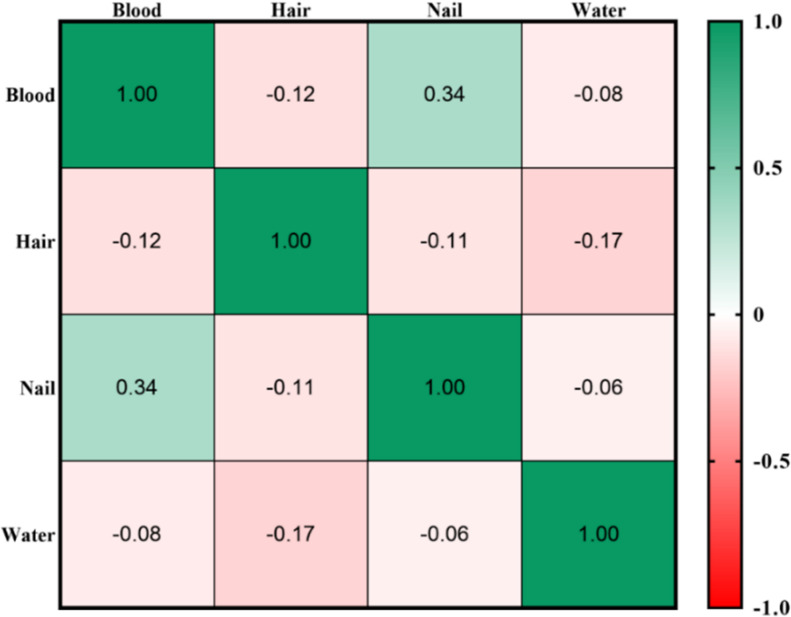
Correlation of average arsenic in water with average arsenic in blood, hair and nails with the help of Pearson correlation.

Pearson’s correlation analysis was employed to examine the relationship between the average arsenic levels in the drinking water and the average arsenic levels in the blood, hair, and nails of the participants in the villages of the study region. As depicted in Fig 5, no significant relationship was observed between arsenic levels in drinking water and arsenic levels in hair, nail and blood. However, a significant positive correlation was detected between the concentration of arsenic in blood and the concentration of arsenic in nails within this region.

### 3.5. Health risk assessment

Exposure to arsenic contaminants through drinking water poses a significant public health concern; Therefore, it is imperative to conduct health risk assessments. Due to the lack of information on the carcinogenic and non-carcinogenic effects of arsenic in the drinking water at the sampling location, the average daily dose (ADD) of arsenic was assessed through the ingestion of drinking water. The mean of average daily dose (ADD) indices of arsenic in drinking water by village were presented in Table 5. The daily intake of arsenic through drinking water made the most significant contribution to the average daily dose (ADD) in villages G, D, and A.

**Table 5:** The mean of average daily dose (ADD) (μg/kg/day), hazard quotient (HQ) and carcinogenic risk (CR) indices of arsenic in drinking water by village.

Village	G	U	D	H	A
Mean concentration of As (μg/l)	16.13	5.05	17.51	8.95	17.14
ADD	0.5012	0.1445	0.5004	0.2560	0.4899
HQ	1.6706	0.4815	1.6680	0.8532	1.6331
CR	0.00075	0.000217	0.00075	0.00038	0.00049

**Fig 6:**
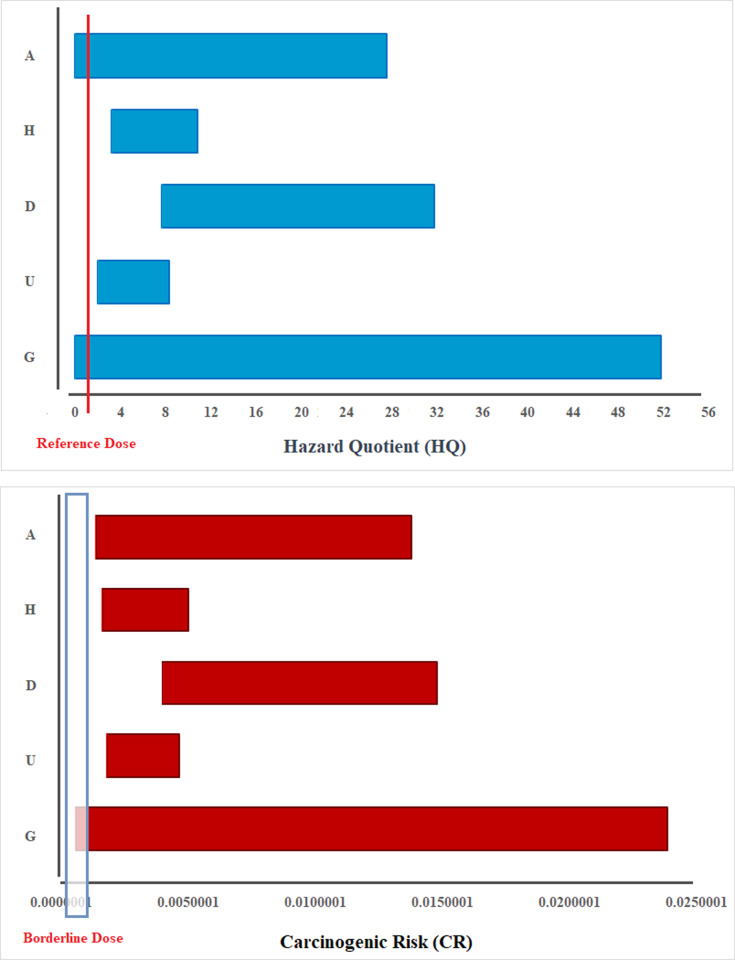
Estimated hazard quotient (HQ) and carcinogenic risk (CR) for drinking water arsenic contaminated by village.

The mean and the range of hazard quotient (HQ) and carcinogenic risk (CR) indices of arsenic in drinking water by village were presented in Table 5 and Fig 6, respectively. These results suggest that the daily intake levels of the examined arsenic were higher than the acceptable threshold (HQ < 1) in G, D and A villages. The non-carcinogenic risk of arsenic ingestion from drinking water in these villages exceeded the safe range, indicating that the high arsenic concentrations could pose a serious health threat to the local population with prolonged consumption.

The cancer risk was ascertained based on the intake levels of inorganic arsenic, which may lead to increased carcinogenic, mutagenic, and teratogenic effects due to inadequate mechanisms for their elimination from the body [33,42]. The estimated cancer risk (CR) due to arsenic in drinking water for inhabitants of different villages is summarized in Table 5. The calculated cancer risk exceeded the safety threshold (1 × 10⁻^4^ as recommended by the US EPA) in all villages, indicating a significant carcinogenic risk from arsenic in the drinking water for residents in these areas. The estimated range of carcinogenic risk (CR) for drinking water contaminated by arsenic in different villages is shown in Fig 6. Accordingly, the widest range of cancer risk (CR) for arsenic was observed in G village, ranging from 9.29 × 10^−7^ to 2.32 × 10^−3^, with an average value of 7.51 × 10^−4^, which is significantly higher than the threshold of 1 × 10^−6^. Similar results were observed in other villages, indicating that the cancer risk from arsenic exposure in the drinking water exceeds the safe level for carcinogenic risk across all sampling areas. Furthermore, the results of this study indicate that the risk of cancer was only lower than 10^−6^ in cases where purchased purified water was consumed (5.9% of samples). Hence, the consumption of drinking water in this region may be significant enough to warrant action under superfund guidelines and could potentially pose detrimental health hazards to the exposed population [33,43].

## 4. Discussion

Routine sampling conducted several months prior in some western Tehran villages showed that arsenic concentrations in rural water sources (drilled wells) exceeded safety limits. This finding prompted the design of a biomonitoring study to assess the health impact of arsenic exposure on residents in the affected areas. The study aimed to measure arsenic levels in both drinking water and biological samples (hair, nails, and blood) of residents. The decision to use biological samples of blood, hair, and nails to assess arsenic exposure is based on their unique characteristics and the comprehensive data they provide regarding exposure levels. Blood is a useful indicator of recent arsenic exposure. Since arsenic is rapidly absorbed into the bloodstream after ingestion, measuring arsenic levels in blood can provide information about recent exposure and the body’s immediate response to arsenic intake [44]. Hair and nail samples reflect long-term arsenic exposure. As hair grows, it incorporates arsenic from the bloodstream, providing a historical record of exposure. This makes hair a valuable sample for assessing cumulative exposure over several months [25].

Similar to hair, nails also accumulate arsenic over time and reflect long-term exposure. Toenails, in particular, can provide an extended timeline of arsenic exposure due to their slower growth rate compared to fingernails. Nail samples offer another non-invasive method for assessing chronic exposure [45]. By analyzing arsenic levels in these three types of biological samples, we can obtain a comprehensive picture of both recent and long-term arsenic exposure among the residents. This multi-faceted approach enhances the accuracy and reliability of our health risk assessment. The results of this study revealed that 47.8% of blood samples exceeded the standard, while the percentages for nail and hair samples were 22.4% and 13.4%, respectively. Moreover, the highest percentage of samples above the standard was found in water samples (67.2%). The preliminary analysis indicated that the average arsenic concentration in the drinking water across all villages was 15.8 μg/liter, surpassing the World Health Organization’s standard [7]. Arsenic intake through ingestion of water is rapidly transported by the blood to the kidneys, liver, lungs, intestines, and skin. Therefore, almost 90% of ingested arsenic is rapidly cleared in the blood within 24 hours. Chronic exposure results in steady-state concentrations of arsenic in both blood and urine, making them valuable biomarkers for assessing past exposure, alongside hair and nails [31]. Arsenic accumulates in hair and fingernails due to its affinity for the abundant sulfhydryl groups in keratin. Therefore, arsenic concentrations in these slow-growing tissues are considered reliable indicators of past exposure. Therefore, the concentration of arsenic higher than the standard limit in the hair and nail samples taken from the residents of the region indicates a long-term exposure to arsenic in the past [46].

The concentration of arsenic in underground water sources is influenced by various factors, including the type and texture of the soil, human activities such as mining and agriculture, climatic conditions, and the level of underground water tables [47]. The west of Tehran is situated in the Urmia-Dokhtar volcanic belt which is considered one of the potentially hazardous regions in terms of heavy metal pollution due to its geological structure [48]. The Urmia-Dokhtar magmatic arc is a long, curved belt of volcanic and plutonic rocks extending approximately 2,000 km from northwest to southeast Iran, primarily composed of andesite and dacite [49]. In this region, arsenic is notably observed in common rock-forming minerals of trachyte, basalt trachyte, dacite, sinodiorite dacite, and basaltic dikes. Furthermore, this region is notable for the mineralization of various elements including gold, silver, copper, lead, zinc, iron, manganese, tin, and tungsten. As these elements are commonly associated with arsenic, it emphasizes the enrichment of rock with arsenic [47]. The presence of the main fault and several sub-faults has activated the region tectonically, creating favorable conditions for the formation of aquifers and providing a suitable opportunity for the development of underground water tables. Despite quantitative and qualitative limitations of water resources, unfavorable climatic conditions, the presence of low-permeability rock formations, and thin alluvial sediment layers, underground water reservoirs have formed in mineral zones along these faults, even though they remain unstable and vulnerable [50].

There are two drivers of pH and electrical conductivity (EC) for large-scale releases of arsenic in the groundwater. Arsenic exists primarily in two oxidation states in groundwater, arsenite (As(III)) and arsenate (As(V)) which is highly dependent on the pH of the groundwater. Higher pH (alkaline conditions) can lead to the desorption of arsenic that was previously adsorbed onto iron and manganese oxides. Under these conditions, arsenite (As(III)) predominates and tends to be more mobile. This is especially relevant in semi-arid and arid environments, where high evaporation rates and mineral weathering contribute to alkaline conditions. The increased solubility of arsenic at high pH levels further exacerbates its elevated concentration in groundwater [8]. The second trigger is related to high electrical conductivity (EC) and total dissolved solids (TDS) values, as well as the oxidation of sulfide minerals and the presence of sulfate (SO_4_) and sodium (Na) sulfate ions. These factors contribute to the development of strongly reducing conditions at near-neutral pH values. These conditions are responsible for the desorption of arsenic from mineral oxides and the reductive dissolution of iron (Fe) and manganese (Mn) oxides, resulting in the release of arsenic. Furthermore, high concentrations of phosphate, bicarbonate, silicate, and possibly organic matter can further enhance the desorption of arsenic due to competition for adsorption sites [8]. Moreover, the high bedrock elevation, shallow groundwater level, reduced thickness of alluvial sediments, and low permeability and mixing due to the fine nature of sediments in the study region contribute to a decrease in water storage quality [51]. On the other hand, decreased rainfall and increased drought due to climate change, along with rising rate of migration to this region and intensified water extraction from the regional aquifer, have reduced the volume of water stored in the aquifer [52]. This decline in groundwater storage has led to an increase in the concentration of pollutants such as arsenic in the water. Furthermore, the fine-grained texture of the sediments significantly reduces the potential for aquifer mixing, infiltration, and replenishment from regional water sources, which in turn exacerbates pollutant concentrations [53].

Arsenic, can naturally occur in groundwater without any anthropogenic contamination. In Bangladesh, over 50 million people are exposed to naturally occurring arsenic concentrations that exceed the World Health Organization’s (WHO) guideline of 10 μg/L [7]. Arsenic speciation was analyzed in urine samples collected from individuals living in an arsenic-contaminated area in Bangladesh in 2012. Their results indicated that the arsenic concentration in the drinking water of 165 married couples ranged from < 0.5 to 332 μg/L, with a median of 55 μg/L. They also found that the urinary concentrations of each arsenic species were significantly correlated with the arsenic concentration in drinking water [54]. In 2014, a cross-sectional study was conducted to assess the concentrations of arsenic and cadmium in urine, blood, and drinking water on 100 residents in both urban and rural communities of Malaysia [55]. Their results revealed significantly higher levels of blood arsenic, urinary cadmium and DNA damage among residents in the rural community. Furthermore, the findings indicated significant correlations between DNA damage and blood arsenic with household income, years of residence, and total daily water consumption among rural residents [55]. Rahman et al. (2009) aimed to assess the association between prenatal arsenic exposure and birth measures such as weight, length, head circumference, and chest circumference. The researchers analyzed data from 1578 mother-infant pairs, which included measurements of urinary arsenic collected at around gestational weeks 8 and 30. The study found that in cases with low levels of arsenic (<100 μg/L in urine), there was a decrease in birth weight by 1.68 g for each 1 μg/L increase of arsenic in urine. However, no negative effect was observed at higher levels of arsenic exposure [56]. In 2018, an agricultural area with arsenic-contaminated shallow acidic groundwater in Thailand was selected and the hair and fingernails of the local people were used as biomarkers to characterize the differences between shallow groundwater drinking and tap water drinking residents. Their results showed that arsenic and heavy metal concentrations in hair and fingernail samples were significantly higher among residents who drank shallow groundwater compared to those who consumed tap water. The regression results revealed that the concentrations of arsenic in the hair samples were associated with various factors, including drinking water source, rate of water consumption, bathing water source, education level, gender, smoking habits, and underlying diseases. While, the factors associated with the concentrations of arsenic in the fingernail samples included drinking water source, occupation, daily work hours, alcohol consumption, gender and pesticide use [57]. Conversely, in the present study, there were no significant correlation in arsenic concentration of biological samples and arsenic in drinking water, type of water drunk, gender, and pesticide use, unlike in other reports. However, some studies have indicated that drinking water may not be the primary contributor to blood and urinary arsenic concentrations [31,58]. One possible explanation for the observed lack of significant correlation between arsenic concentrations in drinking water and biological samples is the limited sample size and low population in the villages included in our study. The relatively small number of biological samples collected may not provide a comprehensive representation of the exposure levels among the entire population [59]. In addition, factors such as individual differences in dietary habits, other environmental exposures, and variations in water source utilization could further obscure any potential relationship between water arsenic levels and biological markers of exposure [60]. For instance, residents might consume food sources with varying levels of arsenic or be exposed to arsenic from other environmental sources (e.g., soil or air) that were not accounted for in our study [61]. These limitations suggest that while our findings indicate elevated arsenic levels in drinking water, the lack of significant correlation with biological samples does not rule out the potential for health risks.

It is unclear why individuals with almost similar lifestyles and arsenic exposure levels differ in their blood arsenic levels and overall response to arsenic exposure [31]. In the present study, we observed that arsenic concentrations in the hair, blood, and nails of smokers were significantly lower than those of non-smokers, despite the well-established exposure to various toxic and carcinogenic metals, including arsenic, through tobacco use [62]. According to the results of a case study carried out in Pakistan in 2011, in lung cancer patients exposed to arsenic, odds ratios indicate evidence of synergism between cigarette smoking and arsenic ingestion through drinking water [31]. This unexpected finding suggests that smokers may metabolize or eliminate arsenic differently, possibly due to interactions between tobacco components and arsenic metabolism pathways. One hypothesis is that certain compounds in tobacco smoke may induce metabolic processes that increase arsenic excretion, leading to lower concentrations in biological samples. Another possibility is that lifestyle or dietary differences, independent of smoking, might influence arsenic levels, particularly given that smoking is often associated with distinct health behaviors. Additionally, the small sample size of smokers in our study (7 individuals) might have impacted the results, and further research with a larger cohort would be needed to confirm and understand the mechanisms underlying these differences.

In this study, we found that arsenic concentrations in blood, hair, and nails were higher in men than in women, although the differences were not statistically significant (p >  0.05). This observation could be attributed to several biological, lifestyle, and environmental factors. Men and women may differ in arsenic metabolism and elimination due to hormonal influences, body composition, and enzyme activity, which may lead to variations in arsenic accumulation. Additionally, men in rural areas are often more engaged in outdoor occupations, such as agriculture, where they might be exposed to arsenic-contaminated dust and water, potentially increasing their overall arsenic burden. Differences in dietary and drinking habits might also play a role, as men may consume more water than women, especially with higher physical activity levels, resulting in elevated arsenic intake. Furthermore, environmental and cultural factors may affect exposure, such as varying uses of well water for household activities. Although these differences were not statistically significant, they suggest a need to explore how gender-specific factors impact arsenic exposure in rural communities.

In addition, there was no association between seafood consumption and blood concentrations of arsenic in this study. However, it was found to be correlated with hair and nail arsenic concentrations. Some authors have also reported that children who consume seafood more frequently exhibit a significantly higher geometric mean blood arsenic concentration compared to those who consume seafood less often [63]. In this study, it was revealed that the mean concentration of arsenic among those who do not use hair removal powder, nail polish, hair dye, or domestic rice was significantly higher. Individuals who do not use hair removal powder or hair dye may have fewer chemical exposures that could otherwise potentially alter or mask the absorption and deposition of arsenic in hair and nails. This reduced interference might result in a higher observed concentration of arsenic. On the hand the absence of nail polish, which could create a barrier and affect the deposition of arsenic, might also result in higher arsenic levels in the nails of non-users. The significant difference in arsenic concentration related to rice consumption may be attributed to varying levels of arsenic in different types of rice. Individuals who consume imported rice might be exposed to lower arsenic levels compared to those consuming domestically grown rice, which might contain higher arsenic levels due to local agricultural practices and soil contamination. Arsenic in hair samples was significantly lower among the non-users of household water purifiers but significantly higher in nail samples. Non-users of household water purifiers might have lower exposure to arsenic in their drinking water, which is reflected in lower arsenic concentrations in their hair. This could be due to the lower availability of arsenic for absorption and subsequent deposition in hair. Conversely, the higher arsenic concentrations in nail samples of non-users might be due to longer-term accumulation of arsenic in nails, which are considered more reliable biomarkers for chronic exposure. Nails grow slowly and may integrate arsenic over a longer period, reflecting chronic exposure levels that hair, with its relatively faster turnover, might not capture as accurately. The absence of a correlation between arsenic concentrations in biological samples and drinking water among residents may be due to the small sample size and limited data available for analysis. This limitation could not be addressed due to the low population of the sampling area. However, the fact that 67% of the residents lack access to safe and arsenic-free drinking water, coupled with the finding that about half of the residents have blood arsenic level exceeding the standard, underscores the urgent need for invention in this area.

Our results indicated that the daily intake of arsenic from drinking water exceeded the reference dose (RfD) value set by the USEPA (0.0003 mg/kg/day) in all sampling locations. In additions, the carcinogenic risk values also were higher the safety level (1 × 10^−4^) recommended by the US EPA. This suggests that drinking water may not be safe for arsenic exposure in these areas. The daily intake of arsenic was lower than the standard only in the sampling sites that consumed purchased purified water with reverse osmosis treatment technology. Several studies have identified significant arsenic exposure to the local population through drinking water which resulted in toxic and carcinogenic health hazard of residents [64,65]. Although a number of studies in some cities of Iran, indicated that the carcinogenic risk of arsenic can be acceptable for both adult and children in dermal and ingestion contact [33,34].

## 5. Conclusion

This study highlights the significant arsenic exposure faced by the population in western Tehran, where 67.2% of water samples exceeded the acceptable arsenic concentration standards. Our findings also demonstrated no significant correlation between arsenic concentrations in drinking water and biological samples. This lack of correlation may be attributed to the limited sample size and low population in the villages. While our results indicate elevated arsenic levels in drinking water, the absence of a significant correlation with biological samples does not exclude the potential for health risks.

To mitigate the health risks associated with carcinogenic arsenic, it is crucial to implement effective intervention, remediation, and control measures. We recommend the establishment of robust drinking water purification programs to protect the health of residents. Additionally, further studies should explore other potential sources of arsenic exposure and develop targeted interventions to address these risks. Employing advanced techniques and methodologies in future investigations will also help overcome the limitations identified in this study. Future research should focus on larger sample sizes and more comprehensive exposure assessments to better identify the sources and consequences of arsenic exposure in these communities.

## Supporting information

S1 File Supporting file (XLSX)
